# *LINC01414/LINC00824* genetic polymorphisms in association with the susceptibility of chronic obstructive pulmonary disease

**DOI:** 10.1186/s12890-021-01579-3

**Published:** 2021-07-07

**Authors:** Xiaoman Zhou, Yunjun Zhang, Yutian Zhang, Quanni Li, Mei Lin, Yixiu Yang, Yufei Xie, Yipeng Ding

**Affiliations:** Department of General Practice, Hainan General Hospital, #19, Xiuhua Road, Xiuying District, Haikou, 570311 Hainan People’s Republic of China

**Keywords:** Chronic obstructive pulmonary disease, *LINC01414/LINC00824*, Polymorphism, Smoking status, Clinical symptom

## Abstract

**Objective:**

Chronic obstructive pulmonary disease (COPD) is a complicated multi-factor, multi-gene disease. Here, we aimed to assess the association of genetic polymorphisms in *LINC01414/ LINC00824* and interactions with COPD susceptibility.

**Methods:**

Three single nucleotide polymorphisms (SNPs) in *LINC01414/LINC00824* was genotyped by Agena MassARRAY platform among 315 COPD patients and 314 controls. Logistic analysis adjusted by age and gender were applied to estimate the genetic contribution of selected SNPs to COPD susceptibility.

**Results:**

*LINC01414* rs699467 (OR = 0.73, 95% CI 0.56–0.94,* p* = 0.015) and *LINC00824* rs7815944 (OR = 0.56, 95% CI 0.31–0.99,* p* = 0.046) might be protective factors for COPD occurrence, while *LINC01414* rs298207 (OR = 2.88, 95% CI 1.31–6.31,* p* = 0.008) risk-allele was related to the increased risk of COPD in the whole population. Rs7815944 was associated with the reduced risk of COPD in the subjects aged > 70 years (OR = 0.29, *p* = 0.005). Rs6994670 (OR = 0.57, *p* = 0.007) contribute to a reduced COPD risk, while rs298207 (OR = 7.94, *p* = 0.009) was related to a higher susceptibility to COPD at age ≤ 70 years. Rs298207 (OR = 2.54, *p* = 0.043) and rs7815944 (OR = 0.43, *p* = 0.028) variants was associated COPD risk among males. Rs7815944 (OR = 0.16,* p* = 0.031) was related to the reduced susceptibility of COPD in former smokers. Moreover, the association between rs298207 genotype and COPD patients with dyspnea was found (OR = 0.50,* p* = 0.016), and rs7815944 was related to COPD patients with wheezing (OR = 0.22,* p* = 0.008).

**Conclusion:**

Our finding provided further insights into *LINC01414*/*LINC00824* polymorphisms at risk of COPD occurrence and accumulated evidence for the genetic susceptibility of COPD.

**Supplementary Information:**

The online version contains supplementary material available at 10.1186/s12890-021-01579-3.

## Introduction

Chronic obstructive pulmonary disease (COPD) is a severely disabling chronic lung disease. COPD is characterized by persistent airflow limitation of respiratory systems due to emphysema and obstructive bronchiolitis [1]. The airflow limitation is caused by the large exposure of lung to harmful particles or gases. At present, the high incidence of COPD exceeds 250 million, which is the third leading cause of death in the world, and it is estimated to cause 4 million deaths every year [2, 3]. In China, COPD caused over 0.9 million deaths is related to several public health problems including pollution, an aging population, and smoking [4, 5]. COPD is a complicated multi-factor, multi-gene disease. Several studies displayed that the occurrence of COPD is associated with various factors such as tobacco smoking, air pollution, pulmonary tuberculosis, occupational exposure and genetic factors [6]. Increasing evidence suggested that genetic polymorphisms exert an important role in COPD occurrence and development [7–9].

Long non-coding RNAs (lncRNAs) is one of the key members of ncRNA family, with greater than 200 nucleotides, participating in the regulators of genetic expression and regulation [10]. Recent study has demonstrated that lncRNAs could contribute to the pathogenesis of respiratory diseases, including COPD [11]. Abnormal expression or function of lncRNAs has been considered to be involved in the development and progression of COPD [12, 13]. Recently, several studies reported some lncRNA gene polymorphisms to the susceptibility of COPD such as *PVT1*, *MiR-146a*, and *nsv823469* [14, 15]. Previously, abnormal expression of *LINC00824* was associated with smoking [16]. However, the contribution of *LINC01414/LINC00824* genetic polymorphisms to COPD predisposition remains unclear.

Here, we genotyped three polymorphisms in *LINC01414/ LINC00824* to assess the genetic association of variants and interactions with COPD susceptibility among the Chinese Han population. Furthermore, the heterogeneity of relationship among subgroups (defined by age, gender and smoking status) and the correlation of selected polymorphisms with clinical symptoms of COPD patients were explored.

## Materials and methods

### Study subjects

A total of 315 COPD patients and 314 healthy controls were enrolled in the present study from Hainan General Hospital. All subjects were ethnic Han Chinese population. COPD patients were diagnosed based on the Global Initiative for Chronic Obstructive Lung Disease (GOLD) [17]. Patients with lung cancer, asthma, tuberculosis, interstitial fibrosis, bronchiectasis, and other respiratory diseases were excluded. Healthy controls who had no cancer history, respiratory diseases, inflammatory or immune diseases were recruited. We collected the demographic and clinical data of all subjects from the questionnaires and medical records. The study was approved by the medical ethics committee of Hainan General Hospital and was in the Declaration of Helsinki. All the subjects signed a written informed consent.

### SNPs genotyping

Peripheral blood samples (5 mL) were collected from each subject into EDTA tubes. A commercially available DNA extraction Kits (GoldMag Co. Ltd, Xi’an, China) was used for the extraction of genomic DNA. Three single nucleotide polymorphisms (SNPs) including rs6994670 and rs298207 in *LINC01414*, rs7815944 in *LINC00824* were selected based on the minor allele frequency (MAF) > 0.05 from 1000 Genomes Project database, Hardy–Weinberg equilibrium (HWE) > 0.05, and the calling rate > 98%. Agena MassARRAY platform (Agena, San Diego, CA, USA) performed the process of genotyping. Primer design (Additional file 1: Table S1) and data management are performed based on corresponding supporting software. About 10% of subjects were repeatedly genotyped for quality control, and the results were consistent.

### Statistical analysis

Sample *t* test or χ^2^ test were used to evaluated the distribution of age and gender between COPD patients and healthy controls. HWE of selected SNPs in controls was detected by a goodness-of-fit χ^2^ test. Logistic analysis adjusted by age and gender were applied to estimate the genetic contribution of selected SNPs to COPD susceptibility by calculating odds ratios (OR) and 95% confidence intervals (CI). Multifactor dimensionality reduction (MDR) analysis was used for analyze gene–gene interaction. Analysis of Variance (ANOVA) was used to evaluate the association between genotypes of *LINC01414*/*LINC00824* variants and clinical characteristics of COPD patients. Data analyses were conducted using SPSS 20.0, PLINK 1.0.7, and MDR software. A *p* value < 0.05 was defined as statistical significance.

## Results

### Participant characteristics.

The participants consisted of 315 cases (239 males and 76 females, 71.9 ± 10.1 years) and 314 controls (237 males and 77 females, 71.2 ± 6.8 years). Table 1 summarized the features of participants, including age, gender, smoking, body mass index (BMI), complication, clinical symptoms (wheezing, dyspnea, chest distress), respiratory rate, pulse rate, forced vital capacity (FVC), forced the first second of expiratory volume (FEV1) and FEV1/FVC. No statistically significant difference in age (*p* = 0.307) and gender (*p* = 0.926) distribution was found.

**Table 1:** Characteristics of patients with COPD patients and controls

Variable	Cases	Controls	*p*
n	315	314	
Age, (mean ± SD) year	71.9 ± 10.1	71.2 ± 6.8	0.307
Gender (male/female), n	239/76	237/77	0.926
Smoking (current/former/never/unavailable), n	83/64/166/2	34/18/118/114	
BMI, (≤ 24 kg/m^2^/ > 24 kg/m^2^/unavailable), n	251/29/35	67/78/169	
COPD with complication (yes/no/unavailable), n	93/174/48		
COPD with wheezing	153/123/39		
COPD with dyspnea	115/166/34		
COPD with chest distress	102/179/34		
respiratory rate, times/min	22.3 ± 2.5		
pulse rate, times/min	86.3 ± 11.7		
FVC, L	2.0 ± 0.7		
FEV1, L	1.1 ± 0.6		
FEV1/FVC, %	51.4 ± 11.8		
GOLD spirometric grade, n (%)			
1	34 (10.8%)		
2	107 (46.7%)		
3	102 (32.4%)		
4	32 (10.2%)		

*COPD* chronic obstructive pulmonary disease, *BMI* body mass index, *FVC* including forced vital capacity, *FEV1* forced the first second of expiratory volume, *GOLD* Global Initiative for Chronic Obstructive Lung Disease

*p* values were calculated by χ^2^ test or the Student’s *t* test

### Correlation of selected polymorphisms with COPD risk

Three SNPs (rs6994670 and rs298207 in *LINC01414*, rs7815944 in *LINC00824*) of the controls were consistent with HWE. The MAF of all the SNPs in this group were > 5% (Table 2). The prevalence of *LINC01414* rs6994670 G-allele frequencies was lower in COPD patients than in controls (OR = 0.73, 95% CI 0.56–0.94,* p* = 0.015).

**Table 2:** The information about the candidate SNPs and the association with COPD in the allele model

Gene	SNP ID	Chr: position	Alleles (Alt/Ref)	MAF		Call rate (%)	HWE			OR (95% CI)	*p*
				Cases	Controls		O(HET)	E(HET)	*p*		
LINC01414	rs6994670	8:65,191,812	G/A	0.214	0.273	99.8	0.393	0.397	0.887	0.73 (0.56–0.94)	0.015*
LINC01414	rs298207	8:65,282,597	A/G	0.229	0.189	98.4	0.320	0.307	0.578	1.27 (0.97–1.68)	0.086
LINC00824	rs7815944	8:129,427,518	G/A	0.265	0.304	99.8	0.390	0.423	0.181	0.83 (0.65–1.06)	0.131

Bold indicate that *p* < 0.05 means the data is statistically significant

*COPD* chronic obstructive pulmonary disease, *SNP* single nucleotide polymorphism, *MAF* minor allele frequency, *HWE* Hardy–Weinberg equilibrium, *O(HET)* observed heterozygotes, *E(HET)* expected heterozygotes

The genetic polymorphisms of selected SNPs were related to COPD susceptibility, as shown in Table 3. Rs699467 in *LINC01414* might be a protective factor for COPD occurrence under the dominant (OR = 0.71, 95% CI 0.51–0.97,* p* = 0.034) and additive (OR = 0.73, 95% CI 0.56–0.95,* p* = 0.018) models. For *LINC01414* rs298207, AA genotype was seen more frequent in COPD-patients compared with GG (OR = 2.87, 95% CI 1.30–6.36,* p* = 0.009) or GG-GA (OR = 2.88, 95% CI 1.31–6.31,* p* = 0.008) genotype. Carriers of GG genotype of rs7815944 in *LINC00824* had a lower frequent in COPD-patients compared with AA genotype (OR = 0.55, 95% CI 0.30–0.99,* p* = 0.047) and AA-AG genotype (OR = 0.56, 95% CI 0.31–0.99,* p* = 0.046).

**Table 3:** Association between candidate SNPs and COPD susceptibility

SNP ID	Model	Genotype	Case	Control	Adjusted by age and gender	
					OR (95%CI)	*p*
*LINC01414* rs6994670	Genotype	AA	194	166	1	
		AG	107	123	0.75 (0.53–1.04)	0.085
		GG	14	24	0.51 (0.25–1.02)	0.056
	Dominant	AA	194	166	1	
		AG-GG	121	147	0.71 (0.51–0.97)	0.034*
	Recessive	AA-AG	301	289	1	
		GG	14	24	0.57 (0.29–1.13)	0.107
	Log-additive	–	–	–	0.73 (0.56–0.95)	0.018*
*LINC01414* rs298207	Genotype	GG	192	201	1	
		GA	94	99	1.00 (0.71–1.41)	0.990
		AA	24	9	2.87 (1.30–6.36)	0.009*
	Dominant	GG	192	201	1	
		GA-AA	118	108	1.15 (0.83–1.60)	0.400
	Recessive	GG-GA	286	300	1	
		AA	24	9	2.88 (1.31–6.31)	0.008*
	Log-additive	–	–	–	1.27 (0.97–1.66)	0.085
*LINC00824* rs7815944	Genotype	AA	168	157	1	
		AG	127	122	0.96 (0.69–1.34)	0.828
		GG	20	34	0.55 (0.30–0.99)	0.047*
	Dominant	AA	168	157	1	
		AG-GG	147	156	0.87 (0.64–1.20)	0.401
	Recessive	AA-AG	295	279	1	
		GG	20	34	0.56 (0.31–0.99)	0.046*
	Log-additive	–-	–-	–-	0.83 (0.65–1.06)	0.128

*p* values were calculated by logistic regression analysis adjusted by age and gender

Bold indicate that *p* < 0.05 means the data is statistically significant

*COPD* chronic obstructive pulmonary disease, *SNP* single nucleotide polymorphism, *OR* odds ratio, *95% CI* 95% confidence interval

### Stratification analysis for the genetic correlation by age, gender and smoking

We also evaluated the contribution of confounding factors (age, gender and smoking status) to the genetic relationship between selected polymorphisms and COPD risk, as listed in Tables 4 and 5. When stratified analysis by age (Table 4), rs7815944 was associated with a reduced COPD risk under the allele (OR = 0.71,* p* = 0.034), genotype (OR = 0.29, *p* = 0.005), recessive (OR = 0.30, *p* = 0.005) and additive (OR = 0.68, *p* = 0.023) models in the subjects aged > 70 years. Rs6994670 was observed to reduce the risk of COPD in the allele (OR = 0.61,* p* = 0.012), genotype (OR = 0.57,* p* = 0.037; and OR = 0.33,* p* = 0.031), dominant (OR = 0.52,* p* = 0.012) and additive (OR = 0.57, *p* = 0.007) models among the subjects with age ≤ 70 years. Rs298207 seem associated to development of COPD (OR = 7.94, *p* = 0.009; and OR = 8.47, *p* = 0.006) at age ≤ 70 years.

**Table 4:** Association between polymorphisms and COPD risk stratified by age and gender

SNP ID	Model	Genotype	Case	Control	OR (95%CI)	*p*	Case	Control	OR (95%CI)	*p*
Age			> 70 years				≤ 70 years			
*LINC01414* rs6994670	Allele	A	297	268	1		198	187	1	
		G	79	84	0.85 (0.60–1.20)	0.356	56	87	0.61 (0.41–0.90)	0.012*
	Genotype	AA	116	102	1		78	64	1	
		AG	65	64	0.94 (0.59–1.47)	0.776	42	59	0.57 (0.33–0.97)	0.037*
		GG	7	10	0.56 (0.20–1.57)	0.271	7	14	0.33 (0.12–0.90)	0.031*
	Dominant	AA	116	102	1		78	64	1	
		AG-GG	72	74	0.88 (0.57–1.36)	0.569	49	73	0.52 (0.31–0.86)	0.012*
	Recessive	AA-AG	181	166	1		120	123	1	
		GG	7	10	0.57 (0.21–1.59)	0.285	7	14	0.42 (0.16–1.12)	0.083
	Log-additive	–			0.85 (0.59–1.23)	0.385			0.57 (0.38–0.86)	0.007*
*LINC01414* rs298207	Allele	G	294	287	1		184	214	1	
		A	78	63	1.21 (0.84–1.75)	0.315	64	54	1.38 (0.91–2.08)	0.126
	Genotype	GG	118	119	1		74	82	1	
		GA	58	49	1.24 (0.77–2.00)	0.379	36	50	0.84 (0.48–1.44)	0.521
		AA	10	7	1.40 (0.50–3.90)	0.522	14	2	7.94 (1.69–37.21)	0.009*
	Dominant	GG	118	119	1		74	82	1	
		GA-AA	68	56	1.26 (0.80–1.98)	0.317	50	52	1.1 (0.66–1.83)	0.726
	Recessive	GG-GA	176	168	1		110	132	1	
		AA	10	7	1.31 (0.47–3.62)	0.602	14	2	8.47 (1.83–39.24)	0.006*
	Log-additive	–-			1.21 (0.83–1.76)	0.311			1.38 (0.91–2.11)	0.134
*LINC00824* rs7815944	Allele	A	278	235	1		185	201	1	
		G	98	117	0.71 (0.51–0.97)	0.034*	69	73	1.03 (0.70–1.51)	0.892
	Genotype	AA	99	82	1		69	75	1	
		AG	80	71	0.90 (0.57–1.42)	0.653	47	51	1.02 (0.60–1.72)	0.951
		GG	9	23	0.29 (0.12–0.68)	0.005*	11	11	1.03 (0.40–2.61)	0.956
	Dominant	AA	99	82	1		69	75	1	
		AG-GG	89	94	0.75 (0.49–1.16)	0.193	58	62	1.02 (0.62–1.67)	0.943
	Recessive	AA-AG	179	153	1		116	126	1	
		GG	9	23	0.30 (0.13–0.70)	0.005*	11	11	1.02 (0.41–2.53)	0.966
	Log-additive	–			0.68 (0.48–0.95)	0.023*			1.02 (0.69–1.49)	0.941
Gender			Male				Female			
*LINC01414* rs298207	Allele	G	367	379	1		111	122	1	
		A	107	91	1.21 (0.89–1.66)	0.226	35	26	1.48 (0.84–2.61)	0.176
	Genotype	GG	147	151	1		45	50	1	
		GA	73	77	0.98 (0.66–1.46)	0.932	21	22	1.06 (0.51–2.18)	0.878
		AA	17	7	2.53 (1.02–6.28)	0.046*	7	2	3.99 (0.77–20.64)	0.099
	Dominant	GG	147	151	1		45	50	1	
		GA-AA	90	84	1.11 (0.76–1.62)	0.582	28	24	1.29 (0.66–2.55)	0.456
	Recessive	GG-GA	220	228	1		66	72	1	
		AA	17	7	2.54 (1.03–6.26)	0.043*	7	2	3.92 (0.77–20.00)	0.101
	Log-additive	–			1.22 (0.89–1.66)	0.218			1.43 (0.82–2.46)	0.204
*LINC00824* rs7815944	Allele	A	362	331	1		101	105	1	
		G	116	141	0.75 (0.56–1.00)	0.052	51	49	1.08 (0.67–1.75)	0.746
	Genotype	AA	134	118	1		34	39	1	
		AG	94	95	0.87 (0.59–1.26)	0.454	33	27	1.41 (0.71–2.81)	0.332
		GG	11	23	0.43 (0.20–0.91)	0.028*	9	11	0.94 (0.35–2.58)	0.912
	Dominant	AA	134	118	1		34	39	1	
		AG-GG	105	118	0.78 (0.54–1.12)	0.179	42	38	1.28 (0.67–2.43)	0.457
	Recessive	AA-AG	228	213	1		67	66	1	
		GG	11	23	0.45 (0.22–0.96)	0.037*	9	11	0.81 (0.31–2.08)	0.655
	Log-additive	–			0.75 (0.56–1.00)	0.052			1.08 (0.68–1.71)	0.753

*p* values were calculated by logistic regression analysis adjusted by age and gender

Bold indicate that *p* < 0.05 means the data is statistically significant

*COPD* chronic obstructive pulmonary disease, *SNP* single nucleotide polymorphism, *OR* odds ratio, *95% CI* 95% confidence interval

**Table 5:** Association between polymorphisms and COPD risk stratified by smoking

SNP ID	Model	Genotype	Case	Control	OR (95%CI)	*p*	Case	Control	OR (95%CI)	*p*	Case	Control	OR (95%CI)	*p*
Smoking			Current				Former				Never			
*LINC00824* rs7815944	Allele	A	123	47	1		97	21	1		239	178	1	
		G	43	21	0.78 (0.42–1.45)	0.438	31	15	0.45 (0.21–0.97)	0.040*	93	58	1.19 (0.82–1.75)	0.361
	Genotype	AA	43	16	1		36	8	1		87	65	1	
		AG	37	15	0.93 (0.40–2.15)	0.867	25	5	1.08 (0.30–3.94)	0.906	65	48	0.99 (0.6–1.62)	0.966
		GG	3	3	0.36 (0.06–2.02)	0.246	3	5	0.17 (0.03–0.96)	0.044*	14	5	1.96 (0.66–5.76)	0.223
	Dominant	AA	43	16	1		36	8	1		87	65	1	
		AG-GG	40	18	0.83 (0.37–1.85)	0.647	28	10	0.68 (0.21–2.14)	0.506	79	53	1.08 (0.67–1.74)	0.751
	Recessive	AA-AG	80	31	1		61	13	1		152	113	1	
		GG	3	3	0.37 (0.07–2.01)	0.250	3	5	0.16 (0.03–0.84)	0.031*	14	5	1.97 (0.68–5.66)	0.211
	Log-additive	–			0.76 (0.39–1.48)	0.413			0.52 (0.22–1.20)	0.124			1.17 (0.79–1.72)	0.443

*p* values were calculated by logistic regression analysis adjusted by age and gender

Bold indicate that *p* < 0.05 means the data is statistically significant

*COPD* chronic obstructive pulmonary disease, *SNP* single nucleotide polymorphism, *OR* odds ratio, *95% CI* 95% confidence interval

In the stratified analysis by gender (Table 4), rs298207 and rs7815944 variants contributed to COPD risk in males. In which, rs298207-AA genotype was seen more frequent in COPD-patients compared with GG (OR = 2.53, *p* = 0.046) or GG-GA (OR = 2.54, *p* = 0.043) genotype among males. Rs7815944 was a protective factor for COPD susceptibility (OR = 0.43, *p* = 0.028; and OR = 0.45, *p* = 0.037) in males.

Stratified analysis by smoking status (Table 5), we found that rs7815944 was associated with a reduced susceptibility of COPD in the allele (OR = 0.45,* p* = 0.040), genotype (OR = 0.17, *p* = 0.044), recessive (OR = 0.16,* p* = 0.031) models among former smokers. However, no significant association of these SNPs with COPD risk in current smokers and never smokers was found.

### Correlation of selected polymorphisms with clinical symptoms in COPD patients

The correlation of selected polymorphisms with clinical symptoms in COPD patients was also assessed, and the results was shown in Table 6. We found the association between rs298207 genotype and COPD patients with dyspnea (OR = 0.50,* p* = 0.016; and OR = 0.56, *p* = 0.029). Moreover, our result displayed that rs7815944 was related to COPD patients with wheezing under the allele (OR = 0.64,* p* = 0.021), genotype (OR = 0.22,* p* = 0.008), recessive (OR = 0.25,* p* = 0.014) and additive (OR = 0.59,* p* = 0.011) models.

**Table 6:** Association of polymorphisms with dyspnea and wheezing in COPD patients

SNP ID	Model	Genotype	COPD with dyspnea				COPD with wheezing			
			Yes	No	OR (95%CI)	*p*	Yes	No	OR (95%CI)	*p*
*LINC01414* rs298207	Allele	G	180	242	1		239	179	1	
		A	48	82	0.79 (0.52–1.18)	0.246	65	59	0.83 (0.55–1.23)	0.349
	Genotype	GG	76	90	1		94	71	1	
		GA	28	62	0.50 (0.29–0.88)	0.016*	51	37	1.03 (0.60–1.74)	0.925
		AA	10	10	0.91 (0.34–2.45)	0.852	7	11	0.40 (0.14–1.13)	0.083
	Dominant	GG	76	90	1		94	71	1	
		GA-AA	38	72	0.56 (0.33–0.94)	0.029*	58	48	0.88 (0.54–1.45)	0.615
	Recessive	GG-GA	104	152	1		145	108	1	
		AA	10	10	1.16 (0.44–3.05)	0.768	7	11	0.40 (0.14–1.10)	0.075
	Log-additive	–			0.72 (0.47–1.08)	0.110			0.80 (0.54–1.18)	0.260
*LINC00824* rs7815944	Allele	A	170	242	1		237	169	1	
		G	60	90	0.95 (0.65–1.39)	0.788	69	77	0.64 (0.44–0.93)	0.021*
	Genotype	AA	62	87	1		89	58	1	
		AG	46	68	0.95 (0.57–1.59)	0.843	59	53	0.72 (0.44–1.20)	0.209
		GG	7	11	0.61 (0.21–1.75)	0.358	5	12	0.22 (0.07–0.67)	0.008*
	Dominant	AA	62	87	1		89	58	1	
		AG-GG	53	79	0.89 (0.55–1.47)	0.660	64	65	0.63 (0.39–1.02)	0.059
	Recessive	AA-AG	108	155	1		148	111	1	
		GG	7	11	0.63 (0.22–1.74)	0.369	5	12	0.25 (0.08–0.76)	0.014*
	Log-additive	–			0.86 (0.58–1.29)	0.472			0.59 (0.40–0.89)	0.011*

*p* values were calculated by logistic regression analysis adjusted by age and gender

Bold indicate that *p* < 0.05 means the data is statistically significant

*COPD* chronic obstructive pulmonary disease, *SNP* single nucleotide polymorphism, *OR* odds ratio, *95% CI* 95% confidence interval

### MDR analysis for gene–gene interaction

**Fig. 1:**
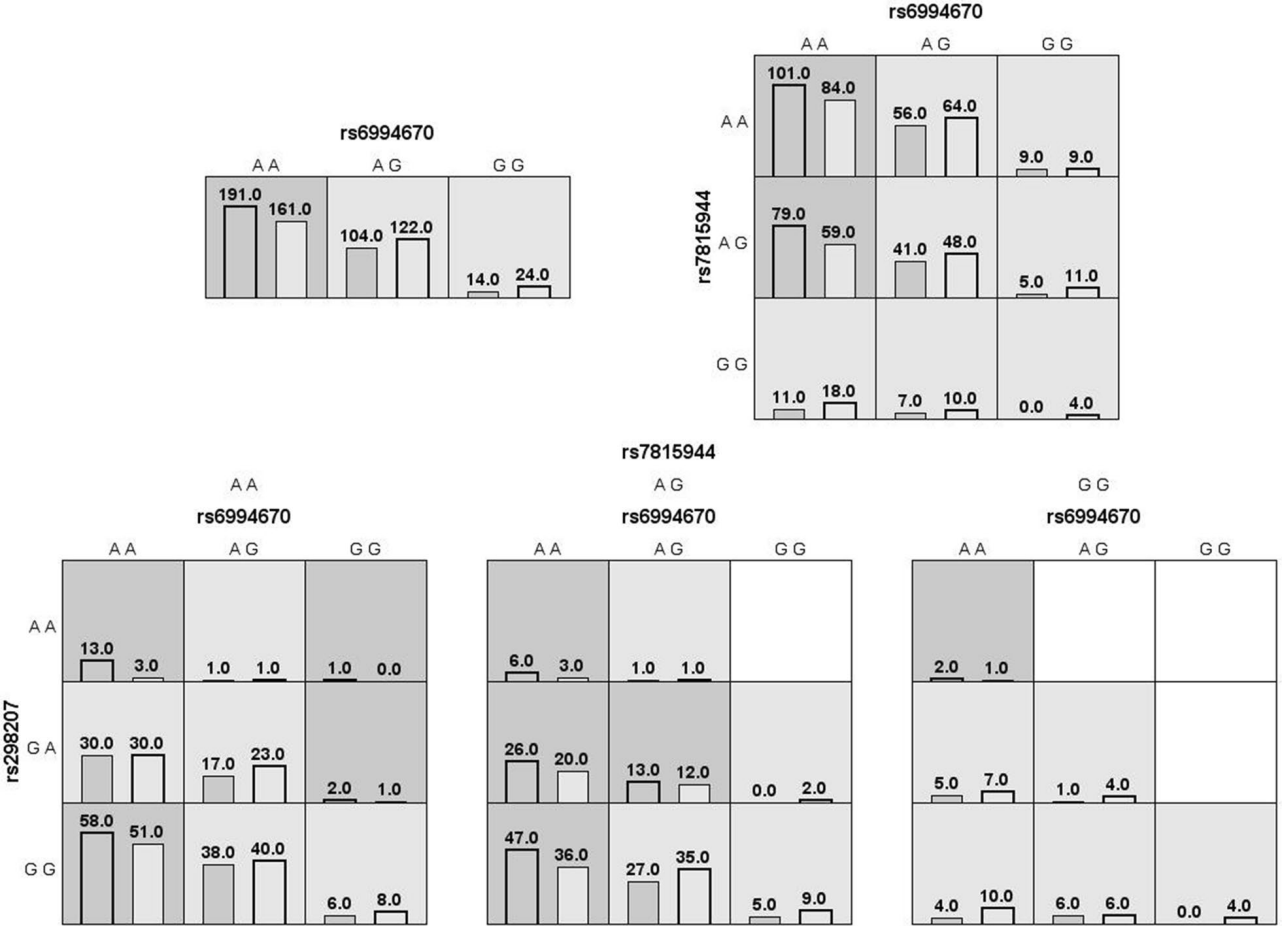
Summary of MDR gene–gene interaction. Each cell shows counts of “case” on left and “control” on right

**Fig. 2:**
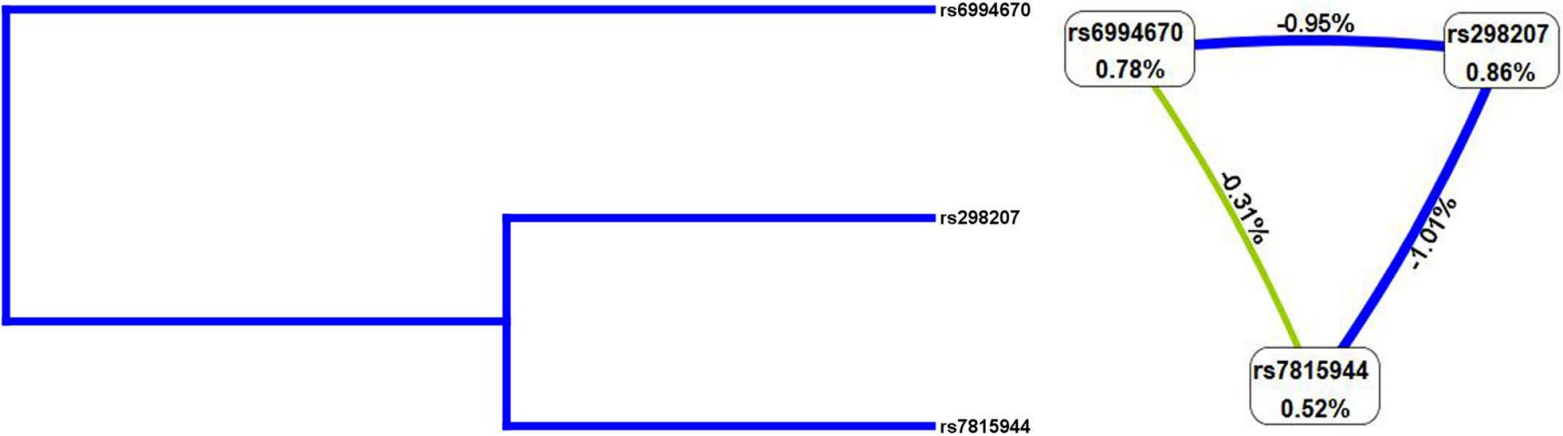
Gene–gene interaction dendrogram and Fruchterman–Reingold

MDR analysis was analyzed to evaluate the contribution of gene–gene interaction to COPD risk. Figure 1 revealed the additive effect between *LINC01414* rs6994670-GG, *LINC01414* rs298207-AA, *LINC00824* rs7815944-GG towards COPD susceptibility. The interactions of these SNPs was displayed as the dendrogram and Fruchterman-Reingold (Fig. 2). Our results demonstrated that *LINC01414* rs6994670 was the best one-factor model for COPD risk (testing accuracy = 0.5468, CVC = 10/10, *p* = 0.0188, Table 7). Furthermore, the two-factor model (*LINC01414* rs6994670 and *LINC00824* rs7815944) was found to be the best multi-loci model for COPD risk (testing accuracy = 0.5518, CVC = 10/10, *p* = 0.0037).

**Table 7:** MDR analysis of gene–gene interaction for COPD risk

Model	Training Bal. Acc	Testing Bal. Acc	CVC	*p*
*LINC01414* rs6994670	0.5468	0.5468	10/10	**0.0188**
*LINC01414* rs6994670, *LINC00824* rs7815944	0.5591	0.5518	10/10	**0.0037**
*LINC01414* rs6994670, *LINC01414* rs298207, *LINC00824* rs7815944	0.5688	0.508	10/10	**0.0012**

*p* values were calculated using χ^2^ tests

Bold indicate that *p* < 0.05 indicates statistical significance

*MDR* multifactor dimensionality reduction, *Bal. Acc.* balanced accuracy, *CVC* cross-validation consistency, *OR* odds ratio, *CI* confidence interval

### The association between selected variants and clinical characteristics of COPD patients

The association between *LINC01414/ LINC00824* SNPs and clinical indicators in COPD patients was assessed, as displayed in Table 8. We found that the genotypes of *LINC00824* rs7815944 was associated with respiratory rate of COPD patients (*p* = 0.022). However, no statistically association was observed on rs6994670 and rs298207 in *LINC01414*.

**Table 8:** Association of clinical characteristics with genotypes of candidate SNPs among COPD patients

Variables	*LINC01414* rs6994670			
	AA	AG	GG	*p*
Respiratory rate, times/min	22.24 ± 2.35	22.49 ± 2.73	21.70 ± 1.57	0.519
Pulse rate, times/min	86.50 ± 11.07	86.86 ± 12.81	78.20 ± 7.63	0.078
FVC, L	2.01 ± 0.71	1.90 ± 0.66	2.03 ± 0.42	0.665
FEV1, L	1.23 ± 0.57	1.20 ± 0.68	1.20 ± 0.41	0.932
FEV1/FVC, %	52.18 ± 11.83	48.55 ± 11.23	57.65 ± 13.46	0.133

Variables	*LINC01414* rs298207			
	AA	GA	GG	*p*
Respiratory rate, times/min	22.15 ± 2.94	22.29 ± 2.43	22.37 ± 2.46	0.914
Pulse rate, times/min	85.05 ± 13.57	87.86 ± 12.44	85.81 ± 11.12	0.357
FVC, L	2.00 ± 0.64	1.89 ± 0.56	2.02 ± 0.75	0.555
FEV1, L	1.28 ± 0.54	1.13 ± 0.48	1.27 ± 0.67	0.384
FEV1/FVC, %	5086 ± 15.95	53.44 ± 210.66	50.27 ± 11.99	0.387

Variables	*LINC00824* rs7815944			
	AA	AG	GG	*p*
Respiratory rate, times/min	22.36 ± 2.5	22.48 ± 2.49	20.78 ± 1.26	**0.022**
Pulse rate, times/min	86.22 ± 11.12	86.32 ± 12.28	87.28 ± 12.86	0.937
FVC, L	1.98 ± 0.61	2.00 ± 0.76	1.84 ± 0.77	0.777
FEV1, L	1.19 ± 0.52	1.22 ± 0.65	1.41 ± 0.81	0.489
FEV1/FVC, %	52.35 ± 11.23	50.34 ± 12.04	50.46 ± 16.01	0.657

*p* values were calculated by Analysis of Variance (ANOVA)

Bold indicate that *p* < 0.05 indicates statistical significance

*COPD* chronic obstructive pulmonary disease, *BMI* body mass index, *FVC* including forced vital capacity, *FEV1* forced the first second of expiratory volume

## Discussion

In our study, rs699467 in *LINC01414* and rs7815944 in *LINC00824* might be protective factors for COPD occurrence, while *LINC01414* rs298207 was associated with the increased risk of COPD in the whole population. Specially, age, gender, and smoking status might contributed to the association of these polymorphisms with COPD risk. Moreover, we found the association between rs298207 genotype and COPD patients with dyspnea, and rs7815944 was related to COPD patients with wheezing. Our findings firstly indicated that *LINC01414*/*LINC00824* polymorphisms might play a role in the occurrence of COPD.

LINC01414, located at chromosome 8q12.3, is a long intergenic non-protein coding RNA 1414. The function of LINC01414 has not been reported. LINC00824, located at chromosome 8q24.21, is also known as LINC01263. Genome-wide association studies reported that *LINC00824* polymorphisms were associated with primary spontaneous pneumothorax and rheumatoid arthritis [18, 19]. Here, we firstly found that rs699467 in *LINC01414* and rs7815944 in *LINC00824* might be protective factors for COPD occurrence, while *LINC01414* rs298207 increased the risk of COPD in the whole population.

The occurrence of COPD is caused by combined effects of genetic background, gender, smoking and an aging population [20]. COPD is the leading causes of disability and death in older people, and significant sex difference can be observed, especially deaths in older men [21]. We also evaluated the contribution of confounding factors (age and gender) to the genetic relationship between selected polymorphisms and COPD risk. Stratified analysis by age, rs7815944 was related to a reduced COPD risk in the subjects aged > 70 years. Rs6994670 was associated with the reduced risk of COPD, while rs298207 might have a higher susceptibility to COPD at age ≤ 70 years. In the stratified analysis by gender, rs298207 and rs7815944 variants were correlated with COPD risk in males. Our results suggested that the genetic contribution of *LINC01414*/*LINC00824* variants to COPD risk was gender- and age-specific. It is generally believed that smoking is the main risk factor for COPD development [22]. Previously, *LINC00824* was higher expression in current smokers compared with former smokers [16]. Stratified analysis by smoking status, we found that rs7815944 was associated with the reduced susceptibility of COPD in former smokers but not current smokers. These hinted that *LINC00824* might have an important role in the COPD pathogenesis. The potential function of rs7815944 is unknown. We speculate that the biological function of rs7815944 may be involved in affecting the expression of I *LINC00824*, which needed to be further studied.

Several limitations in our study is unavoidable. First, the participants were Chinese Han population from a single center (the same hospital), therefore, the selection bias was inevitable and our results are not representative of other ethnic groups. Second, we only analyzed three SNPs in the *LINC01414/LINC00824* gene, and other polymorphisms and other lncRNA genes were not considered. Third, the functional effects of *LINC01414/LINC00824* variants in the pathogenesis of COPD were not explored.

## Conclusion

In summary, we found that *LINC01414* rs699467 and *LINC00824* rs7815944 were associated with lower prevalence of COPD, while *LINC01414* rs298207 was associated with the increased risk of COPD in the Chinese Han population. Our finding provided further insights into *LINC01414/LINC00824* polymorphisms at risk of COPD occurrence and accumulated evidence for the genetic susceptibility of COPD.

## Supplementary Information

**Additional file 1**. Primers sequence.

## Data Availability

The datasets used or analyzed during the current study are available from the corresponding author on reasonable request.
