# Supplementary material for: LINC01414/LINC00824 genetic polymorphisms in association with the susceptibility of chronic obstructive pulmonary disease
Source: BMC Pulm Med. 2021 Jul 7;21:213. doi: 10.1186/s12890-021-01579-3 (PMC8261955; doi:10.1186/s12890-021-01579-3)
Supplement: Supplementary file 1 — Additional file 1. Primers sequence. [file 12890_2021_1579_MOESM1_ESM.docx]

**Table S1. Primers sequence of PCR and UEP used in this study**

| **Genes** | **SNPs** | **First Primer(5'-3')** | **Second Primer (5'-3')** | **UEP_DIR** | | **UEP SEQ (5'-3')** |
| --- | --- | --- | --- | --- | --- | --- |
| LINC01414 | rs6994670 | ACGTTGGATGTCAGATCAGGTTAATGTGGG | ACGTTGGATGGAGATTAAAGCGGTGAACCC | | F | aGGGGACCTCGGTCAATAA |
| LINC01414 | rs298207 | ACGTTGGATGCAGGCGCATAAAACACTAAC | ACGTTGGATGCAGCATGTCTGGCGTGGAG | | R | ttCGCATAAAACACTAACCGACTTT |
| LINC00824 | rs7815944 | ACGTTGGATGCCGTATTTAACCTGGTTCCC | ACGTTGGATGTAGAATGGCCACAGTACAGG | | F | cCTGTTGGTACTTCCTCCC |

SNP, Single nucleotide polymorphism; UEP, Unextended mini sequencing primer; DIR, direction.
